# The calcineruin inhibitor cyclosporine a synergistically enhances the susceptibility of *Candida albicans* biofilms to fluconazole by multiple mechanisms

**DOI:** 10.1186/s12866-016-0728-1

**Published:** 2016-06-18

**Authors:** Wei Jia, Haiyun Zhang, Caiyun Li, Gang Li, Xiaoming Liu, Jun Wei

**Affiliations:** Ningxia Key laboratory of Clinical and Pathogenic Microbiology, the General Hospital of Ningxia Medical University, Yinchuan, Ningxia 750004 China; Center of Laboratory Medicine, the General Hospital of Ningxia Medical University, Yinchuan, Ningxia 750004 China; The First People’s Hospital of Mudanjiang City, Mudanjiang, Helongjiang 157011 China; Department of Laboratory Medicine, College of Clinical Medicine, Ningxia Medical University, Yinchuan, Ningxia 750004 China

**Keywords:** *Candida albicans*, Biofilm, Calcineurin inhibitor, Cyclosporine A, Fluconazole

## Abstract

**Background:**

Biofilms produced by *Candida albicans* (*C. albicans*) are intrinsically resistant to fungicidal agents, which are a main cause of the pathogenesis of catheter infections. Several lines of evidence have demonstrated that calcineurin inhibitor FK506 or cyclosporine A (CsA) can remarkably enhance the antifungal activity of fluconazole (FLC) against biofilm-producing *C. albicans* strain infections. The aim of present study is thus to interrogate the mechanism underpinning the synergistic effect of FLC and calcineurin inhibitors.

**Results:**

Twenty four clinical *C. albicans* strains isolated from bloodstream showed a distinct capacity of biofilm formation. A combination of calcineurin inhibitor CsA and FLC exhibited a dose-dependent synergistic antifungal effect on the growth and biofilm formation of *C. albicans* isolates as determined by a XTT assay and fluorescent microscopy assay. The synergistic effect was accompanied with a significantly down-regulated expression of adhesion-related genes *ALS3*, hypha-related genes *HWP1*, ABC transporter drug-resistant genes *CDR1* and *MDR1*, and FLC targeting gene, *encoding sterol 14alpha-demethylase* (*ERG11*) in clinical *C. albicans* isolates. Furthermore, an addition of CsA significantly reduced the cellular surface hydrophobicity but increased intracellular calcium concentration as determined by a flow cytometry assay (*p* < 0.05).

**Conclusion:**

The results presented in this report demonstrated that the synergistic effect of CsA and FLC on inhibited *C. albicans* biofilm formation and enhanced susceptibility to FLC was in part through a mechanism involved in suppressing the expression of biofilm related and drug-resistant genes, and reducing cellular surface hydrophobicity, as well as evoking intracellular calcium concentration.

## Background

The infection of *Candida albicans* (*C. albicans*) continues to be a major cause of high mortality among immunocompromised and hospitalized patients, and the bloodstream *Candida* infection has been listed as the third most common causes of nosocomial bacteremia and the most common etiologic agent of fungal-related biofilm infection [1, 2]. With an ability to form biofilm seen in the most microorganisms, a formation of *C. albican* biofilm not only provides a protection from environmental stress, but it also allows a horizontal transfer of genes that potentially encode antibiotic resistance, sequentially enhances the resistance of microorganisms to an antimicrobial agent by up to 1000-fold greater than that needed for a treatment of their planktonic counterparts [3, 4].

Fluconazole (FLC) is a member of the azole class, organic compounds posses a five-membered heterocyclic ring with two double bonds, which is the most commonly used first-line agent in the prevention and treatment for patients with candidemia or suspected invasive candidiasis, through a mechanism by which the FLC is able to functionally target encoding sterol 14alpha-demethylase (ERG11), an essential enzyme in the ergosterol biosynthetic pathway of *C. albican* [5]. However, FLC was found to be ineffective in treatment of *C. albicans* biofilm, and the formation of biofilm has been demonstrated to contribute to the failure of anti-fungal treatment, including FLC and other agents, which has been attributed to a compromise in *C. albicans* cell membrane integrity caused by reduced sterols [6]. Intriguingly, mounting evidence has revealed that the antifungal activity of FLC in *C. albicans* biofilm killing could be synergistically enhanced when it was employed in a combination with some antibiotics or immunosuppressants [7–14]. Among them, the calcineurin inhibitors, such as cyclosporine A (CsA) and FK506 have spurred an increased interest [14–19].

Calcineurin is a Ca^2+^-calmodulin-activated phosphatase, which is a multifunctional regulator with functions in governing fungal stress responses, physiological and cell cycle progression, biofilm formation, antifungal resistance, virulence and pathogenesis, and is essential for *C. albicans* survival during membrane stress [20–23]. Several lines of evidence have uncovered that *C. albicans* was resistant to calcineurin inhibitors of CsA and FK506, despite some fungal species were susceptible to these agents. Notably, a combination of either CsA or FK506 with the fluconazole exhibited a synergistic anti-fungal activity to both of planktonic and biofilm *C. albicans* [14–17, 20, 24]. Particularly, the calcineurin inhibitor CsA was recently found to be able to enhance the susceptibility of biofilm-producing *C. albicans* to fluconazole [24]. These results implied that targeting calcineurin signaling using a combination of calcineurin inhibitor FK506 or CsA and FLC might be a promising antifungal strategy for prevention and treatment of biofilm *C. albicans* infection. However, the underlying mechanism by which a calcineurin inhibitor enhances the susceptibility of *C. albicans* to the most common antifungal agent, FLC has yet been fully understood.

In the present report, we aimed to interrogate the molecular mechanism of calcineurin inhibitor CsA in enhancing the susceptibility of biofilm-producing *C. albicans* to FLC by accessing its impacts on the alterations of the expression of drug-transporters and adhesion associated genes, cellular surface hydrophobicity (CSH) and intracellular calcium ([Ca(2+)]) levels. Our results demonstrated that an addition of CsA led an enhanced susceptibility of *C. albicans* to FLC in part through a mechanism by down-regulating the expression of genes associated to ABC transporter and adhesion, a decrease of CSH and an increased intracellular calcium ([Ca(2+)]) level.

## Results

### Biofilm-producing capacity of clinical *Candida albicans* isolates

**Fig. 1:**
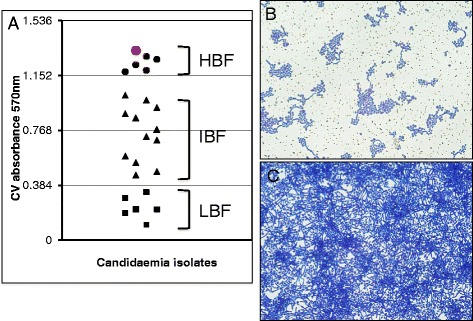
Distinct biofilm forming capacity of clinical *C. albicans* strains isolated from bloodstream. *Candida albicans* isolates were cultured in a 6-well plates for 48 h to allow the maturation of biofilm. The biofilms were then stained with crystal violet, and visualized and imaged under a light microscope. A distinct capacity of biofilm formation was observed among the 24 clinical *C. albicans* isolates. **a** 24 clinical *C. albicans* isolates were categorized in LBF (low biofilm formation capacity) (filled squares), IBF (intermediate biofilm formation capacity) (filled triangles) and HBF (high biofilm formation capacity) (dots). The isolate marked with red dot in HBF group was chosen for further study in this report. **b** A representative image of biofilm produced by a LBF (low biofilm formation capacity) *C. albicans* isolate. **c** A representative images of biofilm produced by a HBF (high biofilm formation capacity) *C. albicans* isolate

In order to evaluate the clinical significance of biofilm in clinical *C. albicans* infection, the capacity of biofilm formation of 24 *C. albicans* clinical isolates were examined. The result showed distinct biofilm-producing capacities of these clinical isolates, which could be categorized into three groups, strain with capacity of low biofilm formation (LBF), intermediate biofilm formation (IBF) and high biofilm formation (HBF), according the absorbance of OD_590nm_ as described as previous report [25]. 6 clinical isolates were fell into LBF with an OD_590nm_ value less than the first quartile (Q1 OD_590nm_ = 0.384), 12 strains in HBF with an OD_590nm_ value greater than the third quartile (Q3 OD_590nm_ = 1.152), and 6 strains could be grouped in IBF with an OD_590nm_ value between Q1 and Q2 in this report (Fig. 1a). Morphologically, patched biofilm with hyphal cells was formed in LBF strains (Fig. 1b), while an intact biofilms could be frequently observed in HBF isolates (Fig. 1c). This data indicated distinct biofilm-producing abilities among clinical *C. albicans* isolates. The isolate with the greatest capacity of biofilm formation (the greatest value of absorbance of OD_590nm_) was selected for further investigation in this study (it was denoted as the red dot in Fig. 1a).

### CsA enhances the susceptibility of clinical biofilm-producing *Candida albicans* to fluconazole

**Fig. 2:**
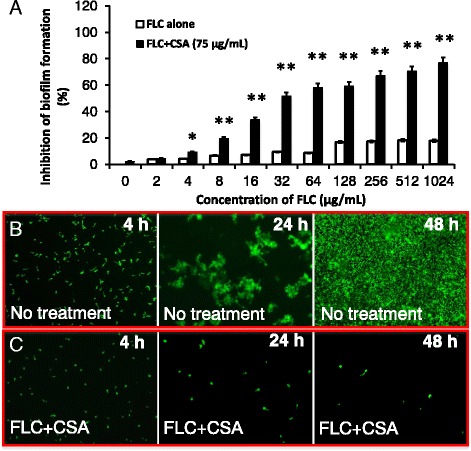
A synergistic effect of calcineurin inhibitor cyclosporine A (CsA) on increasing susceptibility of *C. albicans* isolates to fluconazole. *C. albicans* isolates were cultured in 96-well plates with indicated concentration of FLC or a combination of the indicated concentration of FLC and 75 μg/mL of CsA for 24 h or 48 h. The inhibition of fungi growth was accessed in terms of a XTT assay and fluorescent microscopy. **a** CsA significantly enhances FLC-induced inhibition of the growth of *C. albicans* biofilm-producing isolates at 24 h post-incubation, as compared with FLC alone. Compared with FLC alone group, *: *p* < 0.05; **: *p* < 0.01. Data in A represented the mean ± SD from three independent triplicated experiments (*N* = 9). **b**, **c**. Representative images of biofilm formation of a HLF *C. albicans* isolate for culturing at indicated time, as determined by a fluorescent microscopy assay. **b**. Untreated group. **c**. FLC+CsA treated group. In comparison with the untreated group (**b**), a combination of 75 μg/mL of CsA and 32 μg/mL of FLC dramatically inhibited the cell growth and biofilm formation (**c**)

Calcineurin inhibitors, such as FK506 and CsA have been evidenced to enhance the susceptibility of *C. albicans* to azole agents [14, 17, 18]. In line with these findings, an addition of 75 μg/mL of CsA with FLC significantly led an enhanced susceptibility of clinical biofilm-producing *C. albicans* to FLC by 8- 32-fold over the FLC or CsA alone in the 6 HBF isolates (*p* < 0.01) (Fig. 2a), although FLC alone also showed an insignificant inhibition (<20 %) of biofilm growth of *C. albicans* at concentration of 1024 μg/mL (Fig. 2a). In addition, CsA alone also exhibited a moderate ability to inhibit of biofilm growth (<10 %) of *C. albicans* at concentration of 300 μg/mL (Data not shown). Morphological analysis using fluorescent microscopy further revealed that a combination of 32 μg/mL of FLC and 75 μg/mL of CsA was able to inhibit *C. albicans* cell growth and hyphal formation, while the cells could gradually mature to highly filamentous hyphal cells with a multi-dimensional structure when they were cultured in a naïve condition (Fig. 2b, c).

### A combination of FLC and CsA alters *C. albicans* the expression of drug-resistant genes

**Fig. 3:**
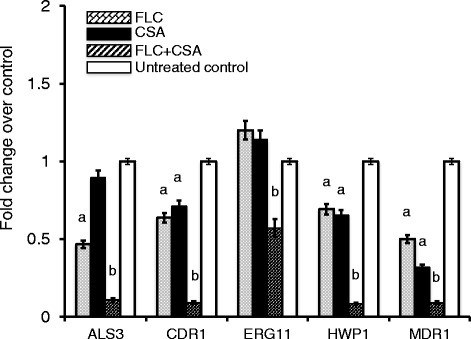
A combination of calcineurin inhibitor CsA and FLC down-regulated the expression of biofilm-related and drug transporter in clinical *C. albicans* isolates. Clinical *C. albicans* isolates were cultured in 6-well plates with 32 μg/mL FLC and 75 μg/mL of CsA alone or in combination for 24 h. The expression of indicated genes was determined by a qRT-PCR assay. The combination of CsA and FLC significantly inhibited the expression of all tested genes, although CsA or FLC along also could moderately down-regulated the expression of these genes. Compared with the untreated group, FLC alone group, a: *p* < 0.05; compared with the CsA or FLC alone group, b: *p* < 0.01. Data represented the mean ± SD from three independent triplicated experiments (*N* = 9)

We next sought the potential molecular mechanism behind the ability of CsA to enhance the susceptibility of biofilm-producing *C. albicans* isolates to FLC, the expression of drug-resistant genes, such as *agglutinin-like sequence 3* (*ALS3*), *hyphal wall protein 1* (*HWP1*), *candlda drug resistance 1* (*CDR1*), *multidrug resistance 1* (*MDR1*) and *ERG11* of cells exposed to 75 μg/mL CsA and 32 μg/mL FLC, was determined by a qRT-PCR assay. Interestingly, a synergistically inhibitory effect on the expression of adhesion-related genes *ALS3*, hypha-related genes *HWP1*, ABC transporter drug-resistant genes *CDR1* and *MDR1*, and FLC targeting gene *ERG11* was observed in the cells treated with a combination of CsA and FLC, which was statistically different as compared with those treated with these agents alone (*p* < 0.05 or 0.01), albeit both CsA and FLC alone also displayed an ability to moderately suppress the expression of above tested gene in biofilm-producing *C. albicans* strains (*p* < 0.05 or *p* > 0.05) (Fig. 3). Of note, both CsA and FLC alone failed to inhibit the *ERG11* gene expression in these clinical biofilm-producing *C. albicans* isolates (Fig. 3). This result imply that the CsA-induced synergistic effect on the enhanced susceptibility to FLC may be in part through a mechanism of down-regulation of the expression of genes associated with cell adhesion, hyphal formation and drug resistance.

### CsA and FLC synergistically reduce cellular surface hydrophobicity of the *C. albicans* strain

**Fig. 4:**
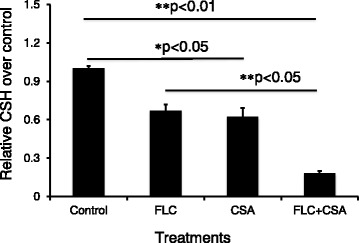
Calcineurin inhibitor CsA and FLC synergistically decrease cellular surface hydrophobicity (CSH) of clinical *C. albicans* isolates. Clinical *C. albicans* isolates were cultured in the presence of 32 μg/mL FLC or 75 μg/mL of CsA alone, or a combination of CsA and FLC for 24 h prior to be used for CSH measurement. The combination of CsA and FLC caused significantly decrease of CSH of *C. albicans* cells, although CsA or FLC along also had a moderate effect on the reduction of CSH. Compared to the untreated control, and treated with CsA or FLC alone groups, **: *p* < 0.01. Data represented the mean ± SD from three independent triplicated experiments (*N* = 9)

A compelling body of studies has demonstrated that the cellular surface hydrophobicity (CSH) is positively correlated with the adhesion and morphological transition, and key processes of *C. albicans* biofilm formation [26–28]. In order to unravel whether a combination of CsA and FLC has an impact on the biofilm formation of *C. albicans*, the CSH of the fungi cells with different treatments was examined. Despite the fungi cells exposed to both CsA and FLC alone showed a significantly decreased CSH relative to the untreated controls (*p* < 0.05), while a combination of CsA and FLC further dramatically inhibited CSH in comparison with the untreated controls (*p* < 0.01) and the cells treated with CsA or FLC alone (*p* < 0.05) (Fig. 4). The evidence suggests that a combination of CsA and FLC has a synergistic effect on the reduction of cellular surface hydrophobicity in these clinical *C. albicans* isolates.

### CsA and FLC synergistically increases intracellular calcium in *C. albicans*

**Fig. 5:**
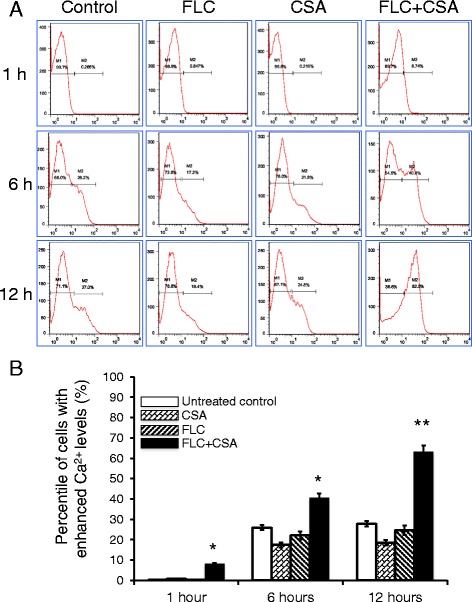
Calcineurin inhibitor CsA and FLC synergistically increase intracellular calcium concentration clinical *C. albicans* isolates. Clinical *C. albicans* isolates were exposed to 32 μg/mL FLC or 75 μg/mL of CsA alone, or a combination of CsA and FLC for 24 h prior to be used for determining intracellular calcium concentration by a flow cytometric assay (FACS). **a** Representative plot images of FACS analysis showed the fraction of cells with high intracellular calcium concentration (M2 fraction) treated with indicated conditions. **b** Quantitative analysis of percentages of cells with high intracellular calcium levels. The result showed that both CsA and FLC alone could reduce intracellular calcium, but a combination of them caused a time-dependently evoked intracellular calcium concentration. Compared with the untreated group of each indicated time point, *: *p* < 0.05; **: *p* < 0.01. Data represented the mean ± SD from three independent triplicated experiments (*N* = 9)

Since the calcium homeostasis is essential in developmental and stress signaling pathways in *C. albicans* [29], the calcium-associated pathways have important implications in key pathogenic steps of this fungal species [30]. Given the fact of that the calcineurin was an important regulator of Ca^2+^ pathways [21], we thus next investigated whether the synergistic antifungal effect of CsA and FLC was associated with calcium homeostasis disturbance, as seen in a previously demonstrated in a combination of minocycline and fluconazole and caused a significant increase of intracellular calcium [13]. As expected, a combination of calcineurin inhibitor CsA and FLC indeed induced a time dependent increase of intracellular calcium, such a fluctuation of Ca^2+^ level was statistically different in comparison with these agents alone, as determined by a FACS assay using Fluo-3/AM indicator staining (*p* < 0.05 for 6 h of treatment, and *p* < 0.01 for 12 h of challenge) (Fig. 5). Intriguingly, the clinical *C. albicans* cells exposed to either CsA or FLC alone exhibited rather lower intracellular levels of Ca^2+^ with slightly changes over time after a 6 h of treatments relative to untreated controls (*p* > 0.05) (Fig. 5). This data clearly indicated that the combination of calcineurin inhibitor CsA and the most commonly used antifungal agent FLC could inhibit the growth of biofilm-producing *C. albicans* clinical isolates by disturbing their intracellular calcium homeostasis.

## Discussion

*Candida albicans* is an important nosocomial infectious agent, and an infection of biofilm-producing *Candida albicans* among immunocompromised patients remains a clinical challenge. In this regard, the use of medical devices such as central venous catheters (CVC’s) and prostheses is a well-known risk factor to induce biofilm formation [31, 32]. Despite a significant advance in our knowledge such as the molecular mechanism of *C. albicans* biofilm formation has been made over the past decade, there is no ideal therapeutic method for bloodstream infections caused by biofilm-producing *Candida albicans* in clinical practice [33].

Accumulating evidences have revealed that a formation of biofilm of *C. albicans* could enhance the resistance of this fungi species to most of the commonly used antifungal agents [1, 34, 35]. Therefore, it is urgent to discover novel antifungal agents or regimens based on new drug targets for the treatment of bloodstream infections, particularly an infection of MDR-biofilm-producing *C. albicans*. With this respect, several lines of evidence have uncovered that a combination of calcineurin inhibitor, such as FK506 and CsA, or antibiotics could synergistically enhance the susceptibility of biofilm-producing *C. albicans* to the first-line antifungal agent FLC [8, 10–14, 17, 18]. In the present report, we also demonstrated that the calcineurin inhibitor CsA had a potential to increase the susceptibility of clinical biofilm-producing *C. albicans* to FLC by suppressing their abilities to form biofilm, and inhibiting the expression of genes related to cell adhesion, hyphal formation and drug-transportators, as well as decreasing cellular surface hydrophobicity and increasing intracellular calcium concentration.

Previous studies have reported that biofilms formed by *C. albicans* strains that isolated from bloodstreams displayed phenotypes associated with drug-resistance and pathogenicity [25]. Therefore, we aimed to morphologically assess biomasses of *C. albicans* clinical isolates using the crystal violet staining, and found distinct capacities of biofilm formations of clinical *Candida albicans* bloodstream isolates, suggesting that the biofilm producing capacity may have an implication of clinical significance. Interestingly, an addition of CsA was verified to be able synergistically increase the susceptibility of these isolates to FLC, along with a down-regulation of the expression of *ALS3, HWP1, CDR1, MDR, ERG11* genes. Among them, *ALS3* are members of the agglutinin-like sequence (*ALS*) gene family that encodes cell-wall glycoproteins [36], and both *ALS* and *HWP1* genes are highly expressed in hyphae and play essential roles in the yeast-to-hypha morphological transition of *C. albicans*, in which the ALS3 contributes cell adhesions, and HWP1 mediates cell substrate and cell-cell interactions in biofilms [37–39]. Therefore, a combination of CsA and FLC-induced down-regulation of these genes might contribute to the anti-biofilm effect by targeting the three known stages for biofilm formation: adhesion to biomaterial surfaces, growth to form an anchoring layer, and morphological transition to form a complex three-dimensional structure [40, 41]. Of note, no alteration or marginal changes of the expression of these genes was found in cells treated with CsA and FLC alone in this study, indicating that the CsA or FLC had limited effect on biofilm growth of *C. albicans*. Equally noteworthy, FLC alone exhibited a limited effect on ERG11 gene expression, which may be in part due to that HBF isolates were more resistant to FLC than LBF strains, and more abundant *ERG11* transcripts were to reported to be detected in FLC-resistant CA strains [42].

The azoles are generally fungistatic agents for treatment and prevention of *C. albicans* infections [43]. However, azole resistant biofilm-producing *C. albicans* infections were frequently observed in clinic settings, which have been attributed to interactions of multiple mechanisms including the alteration of *ERG11* gene expression [44]. *ERG11* gene encodes the 14α-demethylase enzyme which has an effect on ergosterol biosynthesis, and an up-regulated expression of this gene in biofilm *C. albicans* isolates may explain their resistance to azole [45]. In agreement with this notion, exposing biofilm-producing *C. albicans* isolates to a combination of CsA and FLC caused adown-regulation of ergosterol biosynthesis-related gene *ERG11*, which implied an underlying mechanism by which calcineurin inhibitors have potentials to enhance the susceptibility of biofilm-producing *C. albicans* to FLC [14]. It has been previously demonstrated that the highly frequent azole resistance in *C.albicans* strains was in part attributed to an increased efflux of drug mediated mostly by the ATP-binding cassette (ABC) and the major facilitator superfamily (MFS) transporters [46, 47]. In this context, the expression of genes encoding both types of efflux pumps was up-regulated during the course of biofilm formation and development in *C. albicans* [47]. Controversially, a later study by Marchetti et al. suggested that a synergistic antifungal effect of cyclosporine and FLC in *C. albicans* was multidrug efflux transporter genes *CDR1*, *CDR2*, *MDR1* and *FLU1* independent [48]. Inconsistent with this finding, we found that there was a significant down-regulation of efflux transporter genes *CDR1* and *MDR1* in clinical biofilm-producing *C. albicans* isolates treated with a combination of CsA and FLC, suggesting that the CsA-mediated increase of susceptibility of biofilm-producing *C. albicans* to FLC is at least in part through a mechanism by suppressing the expression of these drug transporter genes.

The cell surface hydrophobicity (CSH) of *Candida* species has an implication in the adhesion and biofilm formation of the organisms on epithelial cells or medical device [26, 49], which is also associated with the fungicidal resistance [50–52]. For instance, a FLC resistant *C. tropicalis* strain exhibited a significantly more hydrophobic, greater adherence and higher capacity of biofilm formation on polystyrene surface relative to its parent strain that susceptible to FLC, along with an increased expression of *MDR*1 and *ERG11* genes and enhanced virulence in mice [50]. The discrepancy of CSH and biofilm formation capacity between FLC-susceptible and resistant strains was also recently reported in *C. albicans* cells cultured with different media in presence or absence of FLC [51]. In this regard, *C. albicans* cells dispersed from mature biofilms were more hydrophobic than those dispersed from earlier development stages of biofilms, and *C. albicans* isolates with high capacity of biofilm formation displayed an increased CSH relative to those with lower biofilm formation potential [51]. In agreement with these findings, our result also indicated that calcineurin inhibitor CsA could enhance susceptibility of biofilm-producing *C. albicans* isolates to FLC and prevent cell adhesion on polystyrene surface and biofilm formation (with CSH as the indicator) in part by decreasing CSH.

Ca^2+^ burst is a common cellular response of *C. albicans* cells in response to an environmental stress [53]. It is often along with an activation of calcineurin signaling pathways, in which the calcineurin is required for survival in serum, virulence, and resistance to azole antifungals, in part via its downstream target, Crz1 transcription factor [53–56]. In the present study, a significant Ca^2+^ burst was observed in cells exposed to the combination of CsA and FLC. Of note, either FLC or CsA alone showed an ability to decrease intracellular calcium concentration, however a combination of these two agents had a synergistic effect on increase but not decrease of intracellular calcium ([Ca^(2+)^]) levels. Such CsA-evoked intracellular calcium concentration might disturbed the calcium homeostasis and influenced the cell survival, which may partially explain the potential of CsA to enhance the effectiveness of FLC against the clinical biofilm-producing *C. albicans*. In addition, intracellular calcium was related to biofilm formation. For example, in a study on plant-pathogenic bacterium, *Xylella fastidiosa*, Cruz et al. demonstrated that intracellular calcium played a role in biofilm formation, which was related to the initial surface and cell-to-cell attachment and colonization stages of biofilm establishment, and was depended on functions of fimbrial structures [57].

## Conclusion

In the present study, we provided additional evidences that calcineurin inhibitors (such as CsA) were able to enhance the susceptibility of *C. albicans* clinical biofilm-producing isolates to the most commonly used fungicidal agent, fluconazole (FLC). Mechanistically, CsA could synergistically suppress the expression of adhesion-related genes *ALS3*, hypha-related genes *HWP1*, ABC transporter drug-resistant genes *CDR1* and *MDR1*, and FLC targeting gene *ERG11* in biofilm producing *C. albicans*. In addition, a combination of CsA and FLC also could synergistically reduce cellular surface hydrophobicity (CSH) and increase intracellular calcium concentration in biofilm-producing *C albicans* isolates. Together with other studies, these results clearly suggest a combination of calcineurin inhibitor and fluconazole may prove to be a novel and effective therapeutic option, which warrants for further investigation.

## Methods

### *Candida albicans* strains and culture and identification

*Candida albicans* 1strain SC5314 was purchased from American Type Culture Collection (Mannasas, VA, USA). 24 FLC sensitive *C. albicans* clinical strains were isolated from bloodstream samples and collected from the department of laboratory medicine of the General Hospital of Ningxia Medical University between September 2014 and January 2015, which were identified by harnessing the VITEK-2 COMPACT fully automated microbiological system. The *C. albicans* strains were routinely grown in YPD liquid medium (20 g of glucose per liter, 10 g of yeast extract, 20 g of peptone) at 30 °C with 5%CO_2_ atmosphere [58]. All strains had normal and comparable growth rates. Human blood samples were collected with a protocol approved by the Ethic Committee for the Conduct of Human Research at Ningxia Medical University (NXMU-2016-092). Written consent was obtained from every individual according to the Ethic Committee for the Conduct of Human Research protocol.

### Characterization of *Candida albicans* biofilm formation

*Candida albicans* cells were grown in YPD overnight at 37 °C and resuspended in RPMI buffered with HEPES at a concentration of 1.0 × 10^6^ cells/mL prior to be applied for biofilm formation culturing. The biofilm model was established using a method described in a previous study [59]. Briefly, an 100 μL of above cell suspension was seeded in a flat-bottomed 96 well plates with and incubated at 37 °C at with 5%CO_2_ atmosphere for 24 h or until formation of mature biofilms, and biomass of each isolate was assessed in terms of the crystal violet (cv) assay by determining the distribution of biomass using the value of OD_570nm_ as previously reported [60]. A *C. albicans* isolate with a less than the 1st quartile (Q1) was grouped as having low biofilm formation (LBF) capacity, and a isolate with a biomass greater than the 3rd quartile (Q3) was considered isolates with high biofilm formation (HBF) ability, and an isolate that lay in between Q1 and Q2 was a deemed strain with intermediate biofilm formation (IBF Q2) potency (Fig. 1a) [25]. After the culturing or treatment, harvested the cells by washing and scratching off from the culture wells, the cell suspension was then centrifuged for harvesting cell pellet.

### Test of antifungal susceptibility of biofilm-producing *C. albicans* isolates

The antifungal susceptibility of biofilm-producing C. albicans was ascertained by determine minimum inhibitory concentration (MIC) of fungal cells on 24 h preformed biofilms, as previously described in flat-bottomed, 96 well microtitre plates [60]. The MIC was determined at 80 % inhibition of fungal cells using an XTT (2,3-bis(2-methoxy-4-nitro-5-sulfo-phenyl)-2H-tetrazolium-5-caboxanilide) metabolic reduction assay [61, 62]. The tested range of concentrations of agents was 2 μg/mL to 1024 μg/mL for FLC, and 9.3 μg/mL to 300 μg/mL for CsA. Combinations of these two agents were prepared in a chequerboard format as previously reported [63]. All *C. albicans* strains were tested in duplicate for three independent experiments.

### Fluorescence microscope assay

In order to morphologically observe the formation and integrity of *C. albicans* biofilm, biofilms cultured under different conditions were stained with 50 μg/mL FITC-conA, and imaged using a fluorescent microscopy.

### Quantitative reverse transcriptional PCR (qRT-PCR)

*Candida albicans* cells were homogenized using liquid nitrogen grinding method, and the total RNA was extracted using an RNA purification kit (TaKaRa Biotechnology, Dalian, China). The first strand of cDNA was synthesized by reverse transcription using a commercial RT kit (TaKaRa Biotechnology, Dalian, China). The thermal cycling condition was 94 °C for 4 min as an initial denaturation step, followed by 37 cycles of PCR, consisting of 94 °C for 30 s, 57 °C for 30 s and 72 °C for 30 s. After reacting, a melting curve was evaluated the specificity of the primers. The primer sets for amplifying genes of *agglutinin-like sequence 3* (*ALS3*), *hyphal wall protein 1* (*HWP1*), *candlda drug resistance 1* (*CDR1*), *multidrug resistance 1* (*MDR1*) and *ERG11* were listed in Table 1 [64]. The result was analyzed using 2^-(ΔΔCt)^ [65]. The gene of 18S rRNA was used as an endogenous reference control for normalization the relative expression, and the data was interpreted as fold of changes over the untreated controls. All analysis was carried out on data from three independent experiments with three replicates.

**Table 1:** Primer sequences used in this study

Gene	Primer sequences (5’-3’)	Length
CDR1-F	ACTCCTGCTACCGTGTTGTTATTG	192
R	ACCTGGACCACTTGGAACATATTG	
ERG11-F	AAGAATCCCTGAAACCAA	134
R	CAGCAGCAGTATCCCATC	
MDR-F	GGTGCTGCTACTACTGCTTCTG	226
R	TGATGAAACCCAACACGGAACTAC	
HWP1-F	GCTCAACTTATTGCTATCGCTTATTACA	105
R	GACCGTCTACCTGTGGGACAGT	
ALS3 -F	CAACTTGGGTTATTGAAACAAAAACA	80
R	AGAAACAGAAACCCAAGAACAACCT	
18S rRNA-F	GGATTTACTGAAGACTAACTACTG	144
R	GAACAACAACCGATCCCTAGT	

### Cellular surface hydrophobicity assay

Since a hyphal form of *C. albicans* showed higher affinity for hydrocarbon than the yeast form, and the adherence of these fungus to hydrophobic surfaces increased when its morphology was changed from the yeast form to the hyphal form [66], *C. albicans* cellular surface hydrophobicity (CSH) was assessed using a water-hydrocarbon two-phase assay as described previously [52]. Briefly, *C. albicans* isolates were standardized to 1 × 10^6^ cells/mL in RPMI-1640 and 24 h at 37 °C and washed twice with PBS. *C. albicans* biofilms were scraped off to obtain a cell suspension (OD_600nm_, 1.0 mL in YPD medium). Then, 1.2 mL of cell suspension was transferred into a clean glass tube for each group and overlayed with 0.3 mL of octane. The cell suspension was incubated at 30 °C for 10 min prior the aqueous phase to be measured OD_600nm_ [25]. CSH was calculated using a formula as ([OD_600nm_ of control - OD6_00nm_ of test]/OD600nm of control) × 100 % as previously described [58].

### Detection of intracellular calcium ([Ca^(2+)^]) level

*Candida albicans* biofilms with different treatments were stained with 5 μmol/L of calcium-sensitive indicator Fluo-3/AM (Invitrogen, USA) in light proof at 37 °C for 30 min. The cells were then washed three times with D-Hanks buffer (Invitrogen, USA). The calcium levels were determined by flow cytometry in a FACScan flow cytometer (Becton Dickinson, USA) using a parameter of the excitation/emission wave lengths (485 nm/530 nm) with reading sensitivity level at 8 [10].

#### Statistical analysis

All data were recorded and analyzed by using the WHONET software (version 5.6). The statistical analysis was processed with the Statistical Package for the Social Sciences (SPSS) software (SPSS, version 18.0, Chicago, IL, USA). The changes of MICs for MDR *C. albicans* isolates between FLC or CsA alone and a combination of them were compared with a *t-test* analysis. Data were represented as the mean ± SD. A *p* < 0.05 was defined as a statistical significance.

## Abbreviations

ABC, ATP-binding cassette; ALS3, agglutinin-like sequence 3; *C. albicans: Candida albicans*; CDR1, candlda drug resistance 1; CsA, cyclosporine A; CSH, cellular surface hydrophobicity; CVC, central venous catheters; ERG11,encoding sterol 14alpha-demethylase; FLC, fluconazole; HBF, high biofilm formation; HWP1, hyphal wall protein 1; IBF, intermediate biofilm formation; LBF, low biofilm formation; MDR1, multidrug resistance 1; XTT, 2,3-Bis-(2-methoxy-4-nitro-5-sulfophenyl) -2H-tetrazolium-5-carboxanilide
